# How to extract leucocyte‐poor platelet‐rich plasma using a commercial platelet concentration system developed to obtain leucocyte‐rich platelet‐rich plasma: A pilot study

**DOI:** 10.1002/jeo2.70597

**Published:** 2026-01-04

**Authors:** Theodorakys Marín Fermín, Ayyoub Abood Mohammed Al‐Dolaymi, Jorge Díaz Heredia, Salvador Álvarez Villar, Emmanouil Papakostas, Miguel Ángel Ruiz Ibán

**Affiliations:** ^1^ Clínica Santa Sofía Caracas Venezuela; ^2^ Department of Orthopedic Surgery Military Medical City Hospital Doha Qatar; ^3^ College of Medicine University of Anbar Anbar Iraq; ^4^ Shoulder and Elbow Unit, Orthopaedic Surgery and Trauma Service Hospital Universitario Ramón y Cajal Madrid Spain; ^5^ Facultad de Medicina Alcalá de Henares Madrid Spain; ^6^ Department of Surgery Aspetar Orthopaedic and Sports Medicine Hospital Doha Qatar

**Keywords:** closed system, leucocyte concentration, orthobiologics, platelet concentration, platelet‐rich plasma

## Abstract

**Purpose:**

To develop and characterise a modification of the preparation protocol of a commercial leucocyte‐rich platelet‐rich plasma (LR‐PRP) preparation device (the GPS® III Platelet Concentration System, Biomet, USA) that allows for consistent leucocyte‐poor platelet‐rich plasma (LP‐PRP) extraction using the same components.

**Methods:**

The study included two stages. In the first, the blood of eight healthy adults was processed using the GPS® III Platelet Concentration System (Biomet, USA). Samples were obtained to characterise platelet and leucocyte distribution in the system during the procedure per the manufacturer′s guidelines. In the second, based on the initial results, the standard preparation protocol that yields LR‐PRP was modified to establish a new protocol for obtaining LP‐PRP. To conclude, the newly proposed procedure was validated in 20 individuals, using both the traditional and modified protocols simultaneously.

**Results:**

The characterisation of the commercial system suggested a specific distribution of blood components within the device: most leucocytes were found in the plasma collected through side port #3 (red cap) without waving or shaking the tube, whereas platelets in the sediment between the buoys were released when the tube was waved or shaken. When comparing to the standard protocol, the modified technique consistently yielded significantly lower platelet (MD −205 ×10^3^ platelets/µL, 95% confidence interval [CI], 92.1–317 ×10^3^ platelets/µL, <0.0001) and leucocyte concentrations (MD −19.0 ×10^3^ leucocytes/µL, 95% CI, 14.1–23.9 ×10^3^ leucocytes/µL, *p* < 0.0001) but the obtained LP‐PRP had only 18.1% lower platelet concentration (95% CI, 6.71%–29.4%, *p* < 0.0001) and a relevant 75.3% lower leucocyte concentration (95% CI, 69.6%–81%, *p* < 0.0001).

**Conclusions:**

The proposed modified PRP extraction protocol greatly reduced leucocyte concentrations with a minimal reduction in the platelet concentrations, enabling LP‐PRP preparation with the same commercial device without the need for additional supplies.

**Level of Evidence:**

Level III.

## Graphical abstract

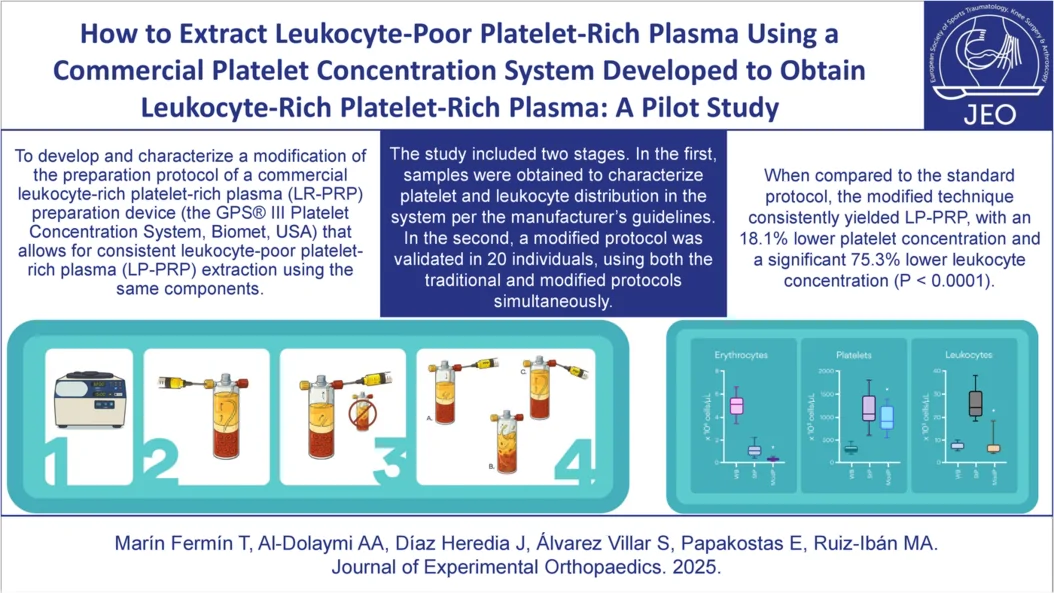

**Abbreviations**
ANOVA: analysis of variance
CI: confidence interval
LP‐PRP: leucocyte‐poor platelet‐rich plasma
LR‐PRP: leucocyte‐rich platelet‐rich plasma
MD: mean difference
PPP: platelet‐poor plasma
PRP: platelet‐rich plasma

## INTRODUCTION

Platelet‐rich plasma (PRP) was first described in 1970 as a plasma portion from autologous whole blood obtained after centrifugation with increased platelet concentrations [23, 27]. Since then, its clinical application has flourished as a regenerative therapy for musculoskeletal pathologies over the decades – including knee osteoarthritis, cartilage injuries, patellar tendonitis, and tennis elbow – and is currently implemented worldwide [2, 14].

PRP composition has been deemed heterogeneous among clinical studies, and despite several attempts to classify and standardise its use, the ideal formulation remains unknown [20]. However, commercial kits may play a critical role in improving the reproducibility of PRP preparations. Most published clinical studies lack relevant information on replicating the implemented PRP preparations, compromising their scientific validity [5, 7, 8, 22]. Thus, a great effort is being made by the orthopaedic community to improve the reporting quality of orthobiologics studies and understand the role of the different PRP components in their efficacy [18, 22, 25, 26, 33].

Platelets and leucocytes are PRP key components, as highlighted by Dohan Ehrenfest et al. in their four‐type classification system: pure platelet‐rich plasma, leucocyte‐ and platelet‐rich plasma, pure platelet‐rich fibrin, and leucocyte‐ and platelet‐rich fibrin [10, 21]. Platelet dose has been linked to improved clinical outcomes, suggesting that achieving a minimum platelet dose in a single‐ or multiple‐injection protocol is paramount [3, 16, 21, 29, 31, 36]. On the other hand, leucocyte dose and composition may have a critical role in managing injuries in different tissues [1, 9, 19, 32]. For example, leucocyte‐rich PRP (LR‐PRP) may be preferred in tendinopathies [12, 19], while leucocyte‐poor PRP (LP‐PRP) in chondropathies, yet the results are still controversial [1, 9, 17, 32].

This study aims to develop and characterise a modification of the preparation protocol of a commercial LR‐PRP preparation device (the GPS® III Platelet Concentration System, Biomet, USA) that allows for consistent LP‐PRP extraction using the same components. The hypothesis was that, after characterising the way the commercial system extracts platelets and leucocytes from whole blood, an adaptation of the procedure could allow for isolated extraction of platelets.

## METHODS

The following experimental study was conducted in the Hospital Universitario Ramón y Cajal between September and October 2024. The Institutional Review Board approved the study (CARACTPRP01‐281/24, v1. Nov2024), and the protocol followed the short‐MIBO guidelines [22].

The study included two stages. In the first stage, the blood of eight healthy adults was processed using the GPS® III Platelet Concentration System (Biomet, USA). Samples were obtained to characterise the platelet and leucocyte distribution in the system during the procedure per the manufacturer′s guidelines. In the second stage, based on the initial results, the standard preparation protocol that yields LR‐PRP was modified to establish a new protocol for obtaining LP‐PRP. To conclude, the newly proposed procedure was validated in 20 individuals, using both the traditional and modified protocols simultaneously.

### Participants

The participants were 28 healthy volunteers who, after signing the appropriate informed consent form and being allowed to ask any questions, underwent a single venipuncture in the median cubital or cephalic vein. Volunteers were considered eligible if they fulfilled the following criteria: (1) were 18–50 years old, (2) had no known comorbidities, and (3) had not taken any non‐steroidal anti‐inflammatory drugs during the last four weeks. Patients were excluded if they (1) had a previous history of blood dyscrasia, (2) were pregnant or (3) were taking any medication during the last four weeks. The volunteers did not receive compensation for their participation in the study. All subjects received written information about the research and their role in it, had the opportunity to ask clarification questions, and later signed a written informed consent form.

All blood and plasma samples were processed immediately after extraction using a Cell‐Dyn Sapphire Haematology Analyser (Abbott, USA) with multi‐angle polarised scatter separation technology and three‐colour fluorescence to obtain leucocyte and platelet counts.

### Stage 1: Characterisation of the commercial PRP concentration system

**Figure 1:**
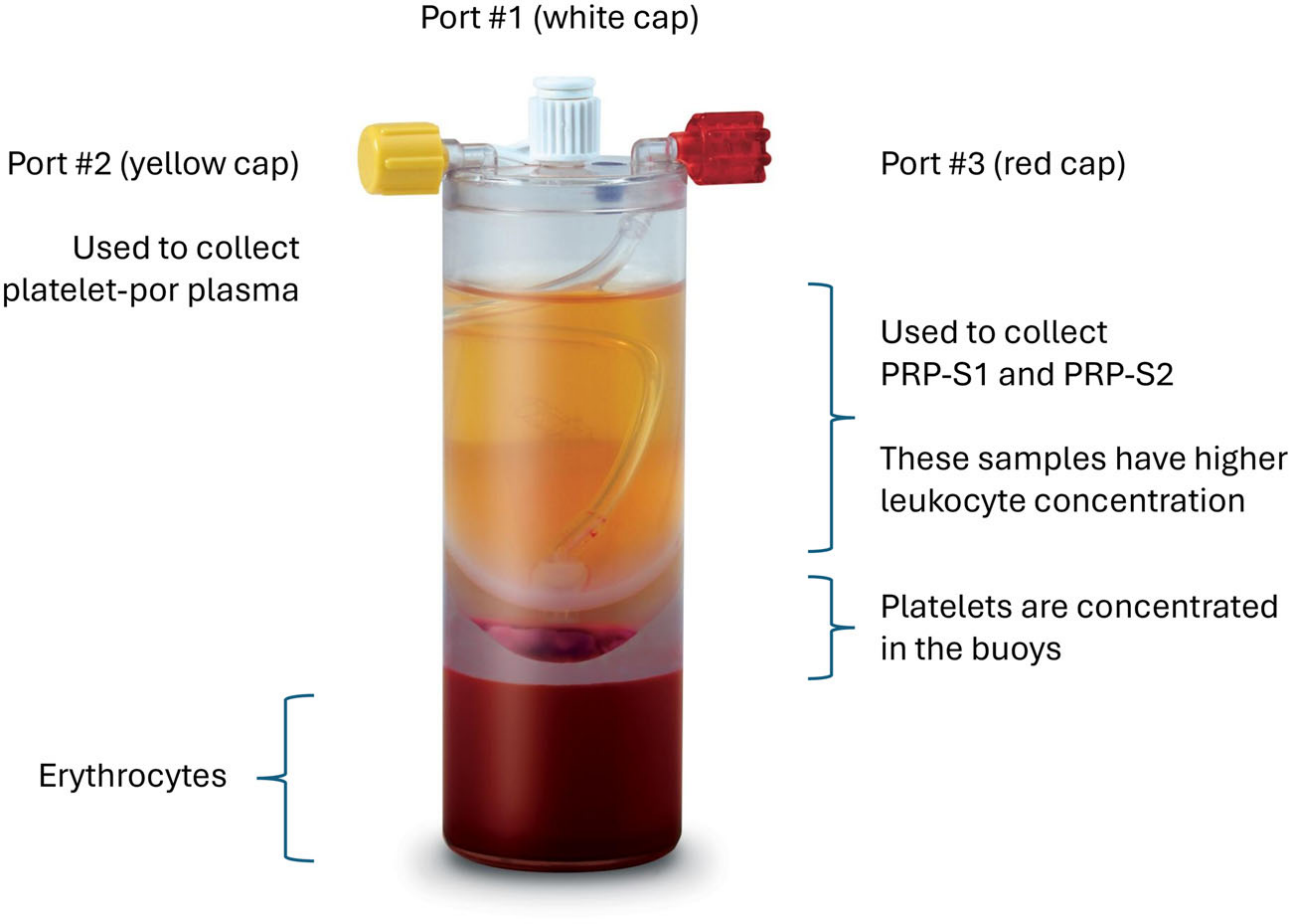
GPS® III Platelet Concentration System (Biomet, USA) anatomy and blood components' distribution.

The GPS® III Platelet Concentration System (Biomet, USA) is a device that allows for the obtention of a LR‐PRP. It is available in 30 mL and 60 mL cartridges, which yield approximately 3 mL or 6 mL of LR‐PRP, respectively (Figure 1).

A single whole blood and four plasma samples were obtained and analysed during the preparation procedure (Table 1) using both 30 and 60 mL cartridges to characterise their mechanism of action.

**Table 1:** Platelet‐rich plasma characterisation of the GPS® III Platelet Concentration System (Biomet, USA).

	30 mL cartridges	60 mL cartridges	30 mL cartridges	60 mL cartridges	30 mL cartridges	60 mL cartridges	30 mL cartridges	60 mL cartridges
**Samples**	**Volume (mL)**		**Erythrocyte [] (x 10** ^**6**^ **cells/µL)**		**Platelet [] (x 10** ^**3**^ **cells/µL)**		**Leucocyte [] (x 10** ^**3**^ **cells/µL)**	
Whole blood	28.52 ± 1.13	48.75 ± 0.56	5.01 ± 0.36	4.90 ± 0.45	217.91 ± 40.78	231.68 ± 48.56	7.14 ± 0.95	7.38 ± 1.22
PPP	11.03 ± 2.05	19.48 ± 1.03	0.11 ± 0.17	0.08 ± 0.13	38.97 ± 28.27	18.94 ± 11.39	0.12 ± 0.13	0.05 ± 0.06
PRP‐S1	1.75 ± 0.20	3.73 ± 0.18	1.87 ± 1.33^a^	1.69 ± 1.18	601.23 ± 209.17^a^	723.38 ± 244.86^a^	30.78 ± 11.07^a^	28.39 ± 10.17^a^
PRP‐S2	1.27 ± 0.26	2.25 ± 0.37	1.01 ± 0.64	0.77 ± 0.61	1034.58 ± 427.61	739.77 ± 217.29	22.71 ± 14.95	15.14 ± 5.80
PRP‐S3	1.88 ± 0.22	3.89 ± 0.17	0.33 ± 0.16	0.26 ± 0.18	1083.54 ± 238.36^b^	1002.30 ± 315.79	6.34 ± 1.14^b^	5.87 ± 2.19^a^, ^b^

*Note*: All data presented as mean ± standard deviation.

Abbreviations: PPP, platelet‐poor plasma; PRP‐S1, platelet‐rich plasma sample 1; PRP‐S2, platelet‐rich plasma sample 2; PRP‐S3, platelet‐rich plasma sample 3.

^a^ Intrasystem (30 mL or 60 mL cartridges) PPP vs. PRP‐S1, or PRP‐S1 vs. PRP‐S2, or PRP‐S2 vs. PRP‐S3 significant differences (*p*<0.05).

^b^ Intrasystem (30 mL or 60 mL cartridges) PRP‐S1 vs. PRP‐S3 significant differences (*p*<0.05).

#### Blood collection

Blood was collected from the antecubital veins of eight individuals using a 30 mL (or 60 mL for the 60‐mL cartridge) syringe loaded with 3 (or 6 mL) calcium citrate according to the GPS® III Platelet Concentration System (Biomet, USA) cartridge. The exact total volume of blood was recorded. The extracted blood was then transferred to GPS® III Platelet Concentration System (Biomet, USA) 30‐ and 60‐mL cartridges and centrifuged at 3200 rpm for 15 min (Biomet Biologics Centrifuge 755VES, Drucker Diagnostics, USA) at room temperature per manufacturer's instructions. Additional blood samples were collected in an EDTA tube for a whole‐blood baseline hemogram.

#### Platelet‐poor plasma extraction

The platelet‐poor plasma portion was extracted from each cartridge after centrifugation through side port #2 (yellow cap) using a 30 mL syringe, and its total volume was recorded. The syringe was gently waved manually, and a 3 mL plasma sample was sent for analytics (PPP). Similarly, a 2 mL (or 4 mL) platelet‐poor plasma sample was reserved for later use.

#### Platelet‐rich plasma sample extraction (PRP‐S1 and PRP‐S2)

First, a 2 mL (or 4 mL) sample was collected through side port #3 (red cap) and sent for analytics (PRP‐S1). Then, without waving or shaking the tube, the remaining volume between the two buoys was extracted from the cartridge. The volumes were recorded and sent for analytics (PRP‐S2). As the manufacturer recommended the waving and shaking procedure to resuspend the platelets that tend to stick to the inner surface of the plastic concentration vessel, this step aimed to determine the maximum number of platelets that could be extracted without stirring or shaking the device.

#### Platelet resuspension and platelet‐rich plasma sample 3 extraction (PRP‐S3)

The 2 mL (or 4 mL) platelet‐poor plasma sample previously collected was injected into the corresponding GPS® III Platelet Concentration System (Biomet, USA) through side port #3 (red cap). The cartridge was firmly shaken for 30 s. Then, all the plasma was extracted with a syringe, the volume was recorded, and samples were allocated for analytics (PRP‐S3).

### Stage 2: Development of a modified protocol. Validation and comparison to the standard protocol

**Figure 2:**
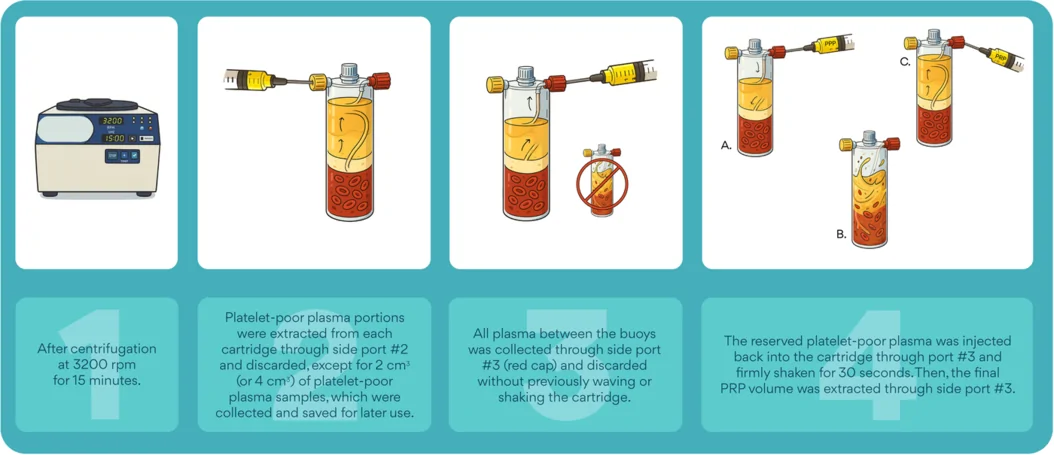
Modified protocol for obtaining leucocyte‐poor platelet‐rich plasma using the GPS® III Platelet Concentration System (Biomet, USA). PPP, platelet‐poor plasma; PRP, platelet‐rich plasma.

The modified protocol for leucocyte‐poor platelet‐rich plasma was conducted as follows: After centrifugation at 3200 rpm for 15 min, platelet‐poor plasma portions were extracted from each cartridge through side port #2 and discarded, except for 2 mL (or 4 mL) of platelet‐poor plasma samples, which were collected and saved for later use. All plasma between the buoys was collected through side port #3 (red cap) and discarded without previously waving or shaking the cartridge. The reserved platelet‐poor plasma was injected back into the cartridge through port #3 and firmly shaken for 30 s. Then, the final PRP volume was extracted through side port #3 (Figure 2).

Twenty healthy individuals had 120 mL of blood extracted in two 60 mL syringes. Each syringe was used independently to prepare the PRP using both the standard and the modified extraction protocols. Whole blood samples were sent for analytics. A 2 cm³ sample from the final PRP preparation for each extraction protocol was sent for analysis. Comparisons were made between the platelet and leucocyte concentrations and yields of each method.

### Statistical analysis

The normality of the quantitative variables was assessed with the Kolmogorov‐Smirnov test. Descriptive statistics were reported as means ± standard deviations. A one‐way analysis of variance (ANOVA) was conducted to assess differences within the samples of the Commercial PRP Concentration System. Comparisons between the standard and modified PRP preparation protocols were performed using paired t‐tests. Statistical significance was defined as *p* < 0.05. All analyses were carried out using GraphPad Prism software, version 10.4.2.

## RESULTS

Twenty‐eight participants (50% males and 50% females), with a mean age of 35.4 ± 15.3 years, were included in the study.

### Stage 1: Characterisation of the commercial PRP concentration system

**Figure 3:**
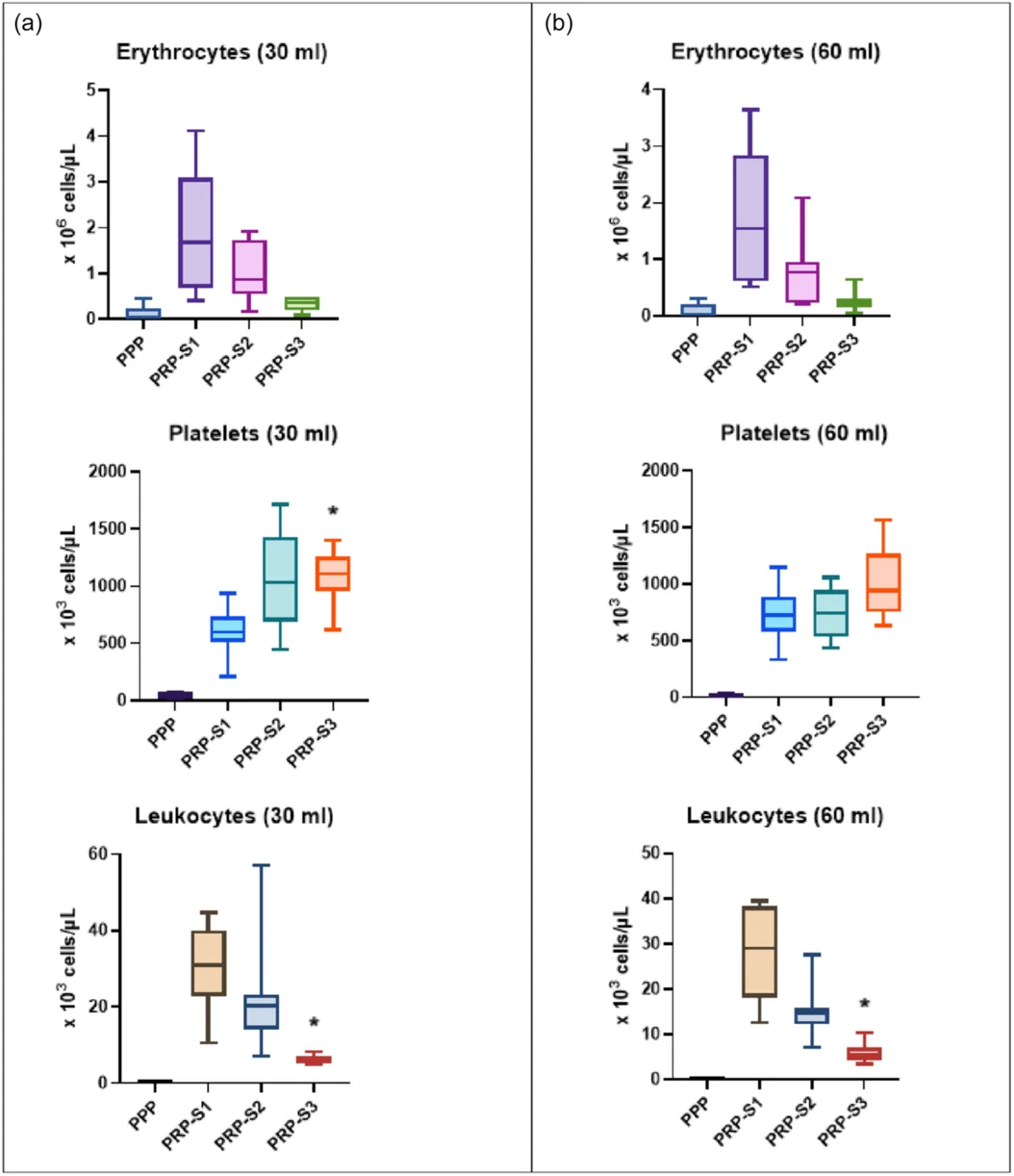
Platelet‐rich plasma characterisation of the GPS® III Platelet Concentration System (Biomet, USA) using: a. 30 mL; and b. 60 mL cartridges. PPP, platelet‐poor plasma; PRP‐S1, platelet‐rich plasma sample 1; PRP‐S2, platelet‐rich plasma sample 2; PRP‐S3, platelet‐rich plasma sample 3. *p < 0.05 (PRP‐S1 vs. PRP‐S3).

PRP‐S1, PRP‐S2 and PRP‐S3 exhibited distinct erythrocyte, platelet, and leucocyte concentration patterns (Figure 3 and Table 1). Compared to PRP‐S1, PRP‐S3 showed significantly lower leucocyte concentrations (30 mL cartridge: mean difference (MD) −24.4 ×10^3^ leucocytes/µL, 95% confidence interval [CI] −12.1 to −36.7 ×10^3^ leucocytes/µL; 60 mL cartridge: MD −22.5 ×10^3^ leucocytes/µL, 95% CI −10.2 to −34.8 ×10^3^ leucocytes/µL) in both cartridge systems (*p* < 0.05). This suggested a specific distribution of blood components within the device: most leucocytes were found in the plasma collected through side port #3 (red cap) without waving or shaking the tube (PRP‐S1), whereas platelets in the sediment between the buoys were released when the tube was waved or shaken. Thus, PRP‐S3 resulted in an LP‐PRP. No intersystem (30 mL vs. 60 mL cartridges) differences were observed.

### Stage 2: Validation of the modified protocol and comparison to the standard protocol

**Figure 4:**
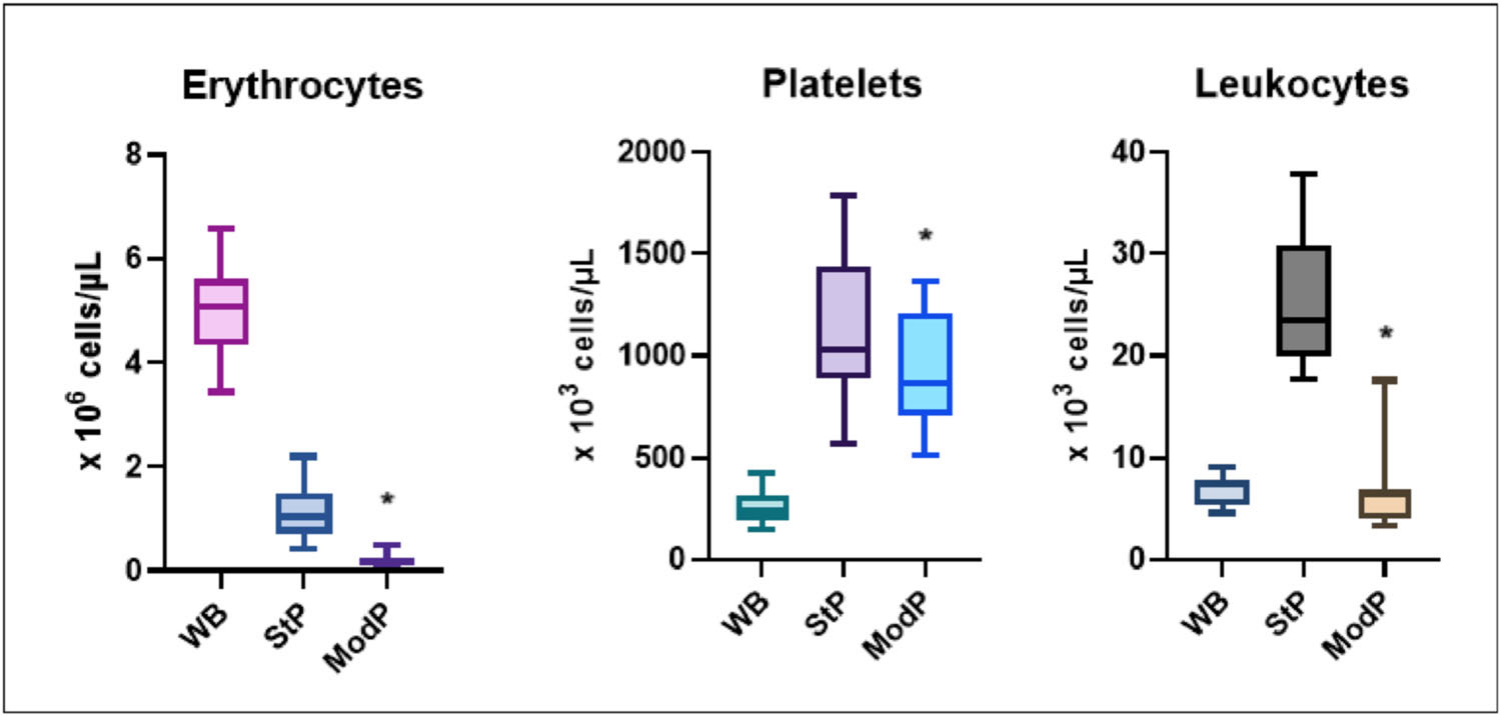
Platelet‐rich plasma characterisation of the GPS® III Platelet Concentration System (Biomet, USA) following the manufacturers' guidelines (StP) versus the proposed modified technique (ModP). *p < 0.05.

When comparing the standard protocol versus the modified protocol using GPS® III Platelet Concentration system (Biomet, USA), the modified technique consistently yielded significantly lower erythrocyte (MD −0.88 ×10^6^ erythrocytes/µL, 95% CI, 0.55–1.22 ×10^6^ erythrocytes/µL, *p* < 0.0001), platelet (MD −205 ×10^3^ platelets/µL, 95% CI, 92.1–317 ×10^3^ platelets/µL, <0.0001), and leucocyte concentrations (MD −19.0 ×10^3^ leucocytes/µL, 95% CI, 14.1–23.9 ×10^3^ leucocytes/µL, *p* < 0.0001; Figure 4 and Table 2). The modified technique resulted in an LP‐PRP with an 18.1% lower platelet concentration (95% CI, 6.71%–29.4%) and a 75.3% lower leucocyte concentration (95% CI, 69.6%–81%) compared to the standard protocol.

**Table 2:** Platelet‐rich plasma characterisation of the GPS® III Platelet Concentration System (Biomet, USA) following the manufacturers' guidelines (standard) versus the proposed modified technique.

Protocol	Erythrocyte**[]**(x10^6^ cells/µL)	Platelet**[]**(x10^3^ cells/µL)	Leucocyte**[]**(x10^3^ cells/µL)
Whole blood	5.02 ± 0.78	254.50 ± 82.79	6.36 ± 1.43
Standard PRP	1.11 ± 0.50	1133.20 ± 347.40	25.27 ± 6.53
Modified PRP	0.22 ± 0.10	928.40 ± 274.08	6.24 ± 3.09
*p* Value^a^	<0.0001	<0.0001	<0.0001

*Note*: All data presented as mean ± standard deviation.

Abbreviation: PRP, platelet‐rich plasma.

^a^ Paired *t*‐test (standard vs. modified PRP)

The standard protocol displayed an increase factor of 4.45 times the baseline platelet concentration with a mean dose of 6.8 billion platelets per 6 mL injection (95% CI, 5.8–7.8 billion platelets per 6 mL injection), while the modified protocol had 3.65 times the baseline with 5.6 billion platelets per 6 mL injection (95% CI, 4.8–6.3 billion platelets per 6 mL injection).

## DISCUSSION

The main finding of the present study is that the modified protocol significantly reduced the quantity of non‐platelet PRP blood components, resulting in a limited reduction in platelet concentration (18.1%) with a relevant reduction of leucocyte concentration (75.3%) compared to the standard protocol. Knowing the specific distribution of PRP components in the system enables the use of either a standard or a modified protocol, allowing for the obtaining of LR‐PRP or LP‐PRP using the same commercially available device.

PRP leucocyte concentration remains a topic of interest and controversy. Controlled laboratory studies have suggested a predominantly anti‐inflammatory effect of LR‐PRP compared to LP‐PRP, as determined by multi‐cytokine profiling, which expresses significantly more IL‐1Ra, IL‐4, and IL‐8. However, MMP‐9 was expressed in higher concentrations, which may suggest a more chondrotoxic effect than LP‐PRP [15]. Similarly, LR‐PRP has been shown to induce more growth factor release and increased tenocyte proliferation than LP‐PRP [19].

Clinically, studies comparing LR‐PRP and LP‐PRP have not shown significant differences in knee osteoarthritis [1, 9, 34]. Two double‐blinded randomised controlled trials comparing three intra‐articular knee injections of LR‐PRP or LP‐PRP revealed no differences in safety, efficacy, subjective and objective clinical outcomes, adverse events, and treatment failure over 12 months [9, 34]. Additionally, a network meta‐analysis by Abbas et al. [1], which included 23 studies (20 randomised controlled trials and 3 prospective cohort studies) involving 2260 patients, found no significant differences in patient‐reported outcomes at a mean follow‐up of 9.9 months. However, the surface under the cumulative ranking probabilities favoured LP‐PRP over LR‐PRP, yet it is irrelevant in clinical practice.

Conversely, LP‐PRP has been shown to improve outcomes when implemented in arthroscopic rotator cuff repair compared to LR‐PRP. A meta‐analysis including nine randomised controlled trials with 540 patients, comparing the effects of LR‐ and LP‐PRP in arthroscopic rotator cuff repair, showed that LP‐PRP significantly improved clinical function and reduced the retear rate, while LR‐PRP did not, except for the VAS score [30]. Moreover, an umbrella review of 11 meta‐analyses on the use of PRP in arthroscopic rotator cuff surgery by Tang et al. [35] revealed that, compared to not using PRP, LP‐PRP improved postoperative pain, retear rate, and postoperative Constant score, while LR‐PRP only improved the postoperative Simple Shoulder Test.

The presented modified preparation protocol offers clinicians an alternative to choosing between LR‐ and LP‐PRP using the same commercial device, without requiring additional supplies. This allows physicians to tailor orthobiologics treatments for specific musculoskeletal injuries. Furthermore, it displays a similar platelet increase factor and platelet dose as reported by Fitzpatrick et al. [13] but lower than previously reported by other authors [6, 11, 24, 26, 28].

The consistency in the decrease of leucocyte concentration among the 20 participants of the present study highlights the potential of the modified protocol for preparing LP‐PRP with this commercial device. Nonetheless, using the modified protocol to deplete leucocytes comes with a marginal loss of almost a fifth of platelets, and this should be weighed considering the increasing evidence on platelet concentration and dose in the efficacy of this orthobiologics therapy [3, 4].

### Limitations

The present study′s main limitation is its underpowered nature, as it represents results from only 20 individuals, whereas normative data typically require a minimum of 120. Additionally, the protocol modification extends beyond the device′s intended use and requires review and approval by regulatory bodies, given the potential for extra manipulation that might necessitate a laminar flow chamber [2]. Future studies should aim for larger multicenter validation and clinical implementation of the presented modified technique.

## CONCLUSION

The proposed modified PRP extraction protocol greatly reduced leucocyte concentrations with a limited reduction in the platelet concentrations, enabling LP‐PRP preparation with the same commercial device without the need for additional supplies.

## AUTHOR CONTRIBUTIONS

*Conceptualisation*: Miguel Ángel Ruiz Ibán. *Methodology*: Jorge Díaz Heredia, Salvador Álvarez Villar and Miguel Ángel Ruiz Ibán. *Validation:* Jorge Díaz Heredia, Salvador Álvarez Villar and Miguel Ángel Ruiz Ibán. *Formal analysis*: Theodorakys Marín Fermín, Emmanouil Papakostas and Miguel Ángel Ruiz Ibán. *Investigation*: Theodorakys Marín Fermín, Ayyoub Abood Mohammed Al‐Dolaymi, Jorge Díaz Heredia, Salvador Álvarez Villar, Emmanouil Papakostas and Miguel Ángel Ruiz Ibán. *Resources*: Theodorakys Marín Fermín and Miguel Ángel Ruiz Ibán. *Data curation*: Jorge Díaz Heredia, Salvador Álvarez Villar and Miguel Ángel Ruiz Ibán. *Writing – original draft*: Theodorakys Marín Fermín, Ayyoub Abood Mohammed Al‐Dolaymi and Miguel Ángel Ruiz Ibán. *Writing – review & editing*: Theodorakys Marín Fermín, Emmanouil Papakostas and Miguel Ángel Ruiz Ibán. *Visualisation*: Theodorakys Marín Fermín and Miguel Ángel Ruiz Ibán. *Supervision*: Emmanouil Papakostas and Miguel Ángel Ruiz Ibán. *Project administration*: Theodorakys Marín Fermín and Miguel Ángel Ruiz Ibán.

## CONFLICT OF INTEREST STATEMENT

The authors have no relevant financial or non‐financial interests to disclose.

## ETHICS STATEMENT

All procedures performed in studies involving human participants were in accordance with the ethical standards of the institutional and/or national research committee and with the 1964 Helsinki Declaration and its later amendments or comparable ethical standards. This study was approved by the Hospital Universitario Ramón y Cajal Institutional Review Board (CARACTPRP01‐281/24, v1. Nov2024). Informed consent was obtained from all individual participants included in the study.

## Data Availability

The data underlying this article are available in the article and its online supplementary material.
